# Supplementary material for: Dynalign II: common secondary structure prediction for RNA homologs with domain insertions
Source: Nucleic Acids Res. 2014 Nov 21;42(22):13939–48. doi: 10.1093/nar/gku1172 (PMC4267632; doi:10.1093/nar/gku1172)
Supplement: SUPPLEMENTARY DATA [file supp_gku1172_nar-02021-z-2014-File012.zip › manual/RNA_class/html/class_two_r_n_a-members.html]

RNAstructure Classes: Member List

- Main Page
- Classes
- Files

- Class List
- Class Hierarchy
- Class Members

# TwoRNA Member List

This is the complete list of members for TwoRNA, including all inherited members.

|  |  |  |
| --- | --- | --- |
| compoundmessage | TwoRNA |  |
| ErrorCodeTwo | TwoRNA | `[protected]` |
| GetErrorCode() | TwoRNA |  |
| GetErrorMessage(const int error) | TwoRNA |  |
| GetErrorMessageString(const int error) | TwoRNA |  |
| GetRNA1() | TwoRNA |  |
| GetRNA2() | TwoRNA |  |
| GetTemperature() | TwoRNA |  |
| rna1 | TwoRNA | `[private]` |
| rna2 | TwoRNA | `[private]` |
| SetTemperature(double temperature) | TwoRNA |  |
| TwoRNA(const char sequence1[], const char sequence2[], bool IsRNA=true) | TwoRNA |  |
| TwoRNA(const char filename1[], const int type1, const char filename2[], const int type2, bool IsRNA=true) | TwoRNA |  |
| TwoRNA() | TwoRNA |  |
| ~TwoRNA() | TwoRNA |  |

---

Generated on Tue Jul 30 10:52:29 2013 for RNAstructure Classes by 
 1.5.7.1
